# Discrepancies of assessments in a RECIST 1.1 phase II clinical trial – association between adjudication rate and variability in images and tumors selection

**DOI:** 10.1186/s40644-018-0186-0

**Published:** 2018-12-11

**Authors:** Hubert Beaumont, Tracey L. Evans, Catherine Klifa, Ali Guermazi, Sae Rom Hong, Mustapha Chadjaa, Zsuzsanna Monostori

**Affiliations:** 1 Research & Clinical Development, Median Technologies, Les deux arcs - 1800 route des crêtes – Bat, B 06560 Valbonne, France; 2 Department of medicine, Hospital of the University of Pennsylvania, Philadelphia, PA 19104 USA; 3 Quantitative Imaging Center (QIC) Boston University School of Medicine, Boston, MA 02118 USA; 4 Department of Radiology, Severance Hospital Yonsei University of Medicine, Seoul, South Korea; 5 Clinical Research, SANOFI, 94400 Vitry-sur-seine, Paris, France; 6 Radiology, National Koranyi Institute of TB and pulmonology, Budapest, H-1121 Hungary

**Keywords:** Response evaluation criteria in solid tumors, Inter-observer variability, Tumor imaging, Small cell lung carcinoma, Phase II

## Abstract

**Background:**

In imaging-based clinical trials, it is common practice to perform double reads for each image, discrepant interpretations can result from these two different evaluations. In this study we analyzed discrepancies that occurred between local investigators (LI) and blinded independent central review (BICR) by comparing reader-selected imaging scans and lesions. Our goal was to identify the causes of discrepant declarations of progressive disease (PD) between LI and BICR in a clinical trial.

**Methods:**

We retrospectively analyzed imaging data from a RECIST 1.1-based, multi-sites, phase II clinical trial of 179 patients with adult small cell lung cancer, treated with Cabazitaxel compared to Topotecan. Any discrepancies in the determination of PD between LI and BICR readers were reviewed by a third-party adjudicator. For each imaging time point and reader, we recorded the selected target lesions, non-target lesions, and new lesions. Odds ratios were calculated to measure the association between discrepant declarations of PD and the differences in reviewed imaging scans (e.g. same imaging modality but with different reconstruction parameters) and selected lesions. Reasons for discrepancies were analyzed.

**Results:**

The average number of target lesions found by LI and BICR was respectively 2.9 and 3.4 per patient (*p* < 0.05), 18.4% of these target lesions were actually non-measurable. LI and BICR performed their evaluations based on different baseline imaging scans for 59% of the patients, they selected at least one different target lesion in 85% of patients. A total of 36.7% of patients required adjudication. Reasons of adjudication included differences in 1) reporting new lesions (53.7%), 2) the measured change of the tumor burden (18.5%), and 3) the progression of non-target lesions (11.2%). The rate of discrepancy was not associated with the selection of non-measurable target lesions or with the readers’ assessment of different images. Paradoxically, more discrepancies occurred when LI and BICR selected exactly the same target lesions at baseline compared to when readers selected not exactly the same lesions.

**Conclusions:**

For a large proportion of evaluations, LI and BICR did not select the same imaging scans and target lesions but with a limited impact on the rate of discrepancy. The majority of discrepancies were explained by the difference in detecting new lesions.

**Trial Registration:**

ARD12166 (https://clinicaltrials.gov/ct2/show/NCT01500720).

## Background

Double interpretation of oncologic images is often considered when an imaging endpoint is used in a clinical trial [1]. Such dual review allows for independent assessment of treatment response. In a clinical trial setting, images may first be read by local investigators (LI) who are generally at the center where patients underwent the trial imaging scans. An independent reader expert, blinded to the treatment assignment and known as the blinded independent central review (BICR) then does a second image evaluation. Discordant interpretations between the two evaluations are common [2]. Reasons for inter-reader variability in lesion measurement have been predominantly documented [3] [4], even though other factors [5] are known to also impact the reliability of therapeutic response assessments. The variability in tumor response assessment may reduce the statistical power required to detect true treatment effect. Tackling inter-reader variability will improve the reliability of treatment evaluations and will enable more reliable comparisons between drug treatments, notably for trials where Progression Free Survival (PFS) is the primary endpoint according to Sridhara et al. [6].

Discordances in radiological interpretation can be observed between readers in different settings (i.e. between LI sites and BICRs (e.g., CRO readers) [7]). However, inter-reader discordances have also been observed when two readers follow the same standard procedure within the same site [8]. In a meta-analysis by Cohen et al. [9] or by Ford et al. [10], the discordance rates were, respectively, ranging 23–46% and 42% among BICRs readers.

In order to address these reading discrepancies, a third reader is generally brought in as an adjudicator. The adjudication rate, defined as the ratio of patients requiring adjudication to the total number of patients in the study, has become an important study metric in clinical trials and is a high-level indicator of clinical trial performance. A low adjudication rate therefore translates into good compliance with the study protocol and procedures and stratified analysis of adjudication rates can be highly informative [11, 12].

The Response Evaluation Criteria In Solid Tumors (RECIST) [13] are a set of rules used to monitor therapeutic response in oncology and that constrain readers to select a given number of target lesions (lesions whose longest axial diameter is larger than 10 mm (non-nodal)), selected for robust repeated measurements. The criteria also require the assessment of non-target lesions (lesions smaller than the 10 mm threshold or too complex for repeated measurements) and the reporting of new lesion appearance. However, RECIST rules carry some subjectivity that represents a potential factor for inter-reader variability. Poor guidance in reporting new lesions, in reporting progression of non-target lesions or in the optimal use of window level to visualize lesions, are factors that may lead to subjective assessments. In addition to RECIST subjectivity issues, imaging-based clinical trials generate a large amount of data for each patient, providing several acquisitions either from the same imaging modality (i.e. CT scan thick vs thin slices, various gaps or fields of view, etc) or from different imaging modalities (CT vs MRI vs PET, etc) and at multiple time points. In addition, imaging scans may display multiple tumors of various sizes across different organs. With such diversity in imaging acquisition and tumor data per patient at each time point, inter-reader data variability appears to be unavoidable. Whether this diversity affects the adjudication rate remains a relevant question.

In this retrospective study, we used data from the ARD12166 Small Cell Lung Cancer trial to analyze several predefined risk factors (see Table 1) likely to trigger adjudications between LI and BICR evaluations for progressive disease. First, we hypothesized that readers selecting different series within the same exam (at baseline or follow-up) were likely to lead to inconsistent measurements between readers [14]. Second, that there would be reading differences in detecting new lesions or in assessing progression of non-target lesions. Third, that the evaluation of the tumor burden (sum of target lesion diameters) per patient [15] can be variable.

**Table 1:** Definition of the different risk factors likely to triggering adjudications. List of terms used as predefined risk factors and their corresponding definitions

Risk factors	Explanation
1.Different scans at baseline	LI and BICR selected scans with different SeriesUID as DICOM Tag. (e.g. same imaging modality but with different reconstruction parameters)
2. Different scan(s) at follow up	LI and BICR selected at least one different scan with different SeriesUID as DICOM Tag during patient follow up. Their measures were extracted from different scans at at least one time point after baseline.
3. Different number of target lesions	Readers selected a different total number of target lesions in the tumor burden.
4. New lesions	If any reader reported one or more lesion(s) that was/were not recorded on the previous time point.
5. Different target Lesions	LI and BICR each selected a tumor burden that does not contain exactly the same lesions. Note: Both readers may have selected the same number of target lesions, however not the same ones.
6. Non-measurable lesions	LI and BICR measured the same Target Lesion but did not have the same perception of lesion boundaries (due to its complexity). We adopted part of RECIST 1.1 definition for non-measurable lesions [24] as *lesions likely to lead to non-reproducible measurements*. The labelling of non-measurable lesions is done on baseline data only.
7. Progressive non-target lesion	A patient is classified as having progressive disease by non-target lesions when either LI, BICR or both declared progression based on non-target lesions. (According to RECIST, *unequivocal progression of non-target lesions* has a very specific definition which is supposed to be an “extremely rare” event.)

Finally, we pointed out the most critical risk factors for this specific disease and in this trial settings. We expect our analysis to help in developing efficient standardized reading solutions.

## Materials and methods

### Analyzed data

Our retrospective study is based on data collected during the ARD12166 trial. ARD12166 was a RECIST 1.1, phase II trial for patients with small cell lung cancer (SCLC). Patients had advanced metastatic disease located mainly in lymph nodes and liver. [16]. Full details about ARD12166 trial and patients are given in annex I and II respectively.

### Analysis procedure

**Fig. 1:**
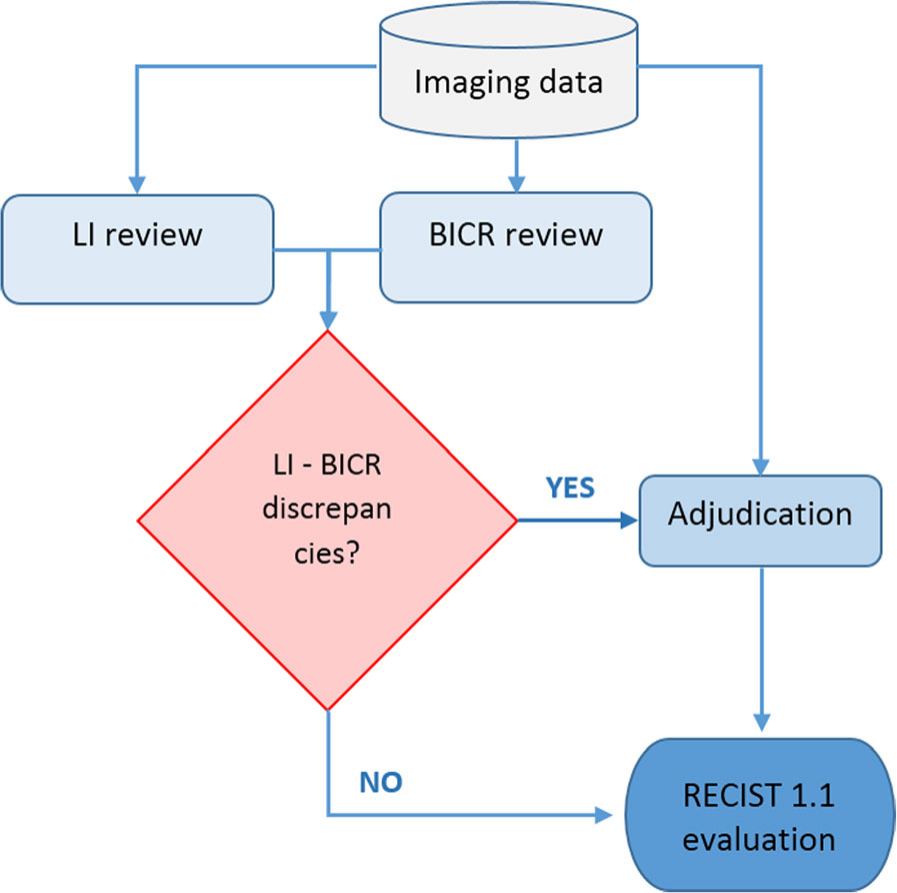
Flowchart of the ARD12166 trial. Using a common database of imaging scans, LI and BICR performed RECIST 1.1 evaluations. In case of evaluations discrepancies, an adjudicator was solicited to perform a third evaluations blinded from previous assessments

The Fig. 1 displays a flowchart from the initial ARD12166 trial that we analyzed. In our study, we restricted the definition of the adjudication rate, to the ratio of patients requiring adjudication for declaring a progressive disease to the total number of patients in the study.

All imaging scans, readers’ notes and measurements from the ARD12166 trial were originally saved into databases. Our retrospective analysis was therefore possible and presented in a similar radiological context as the original study. A software (Median Technologies, Valbonne, France) automatically analyzed the original trial databases to measure:

1. the proportion of patient for which LI and BICR annotated the same scans (same imaging modality parameters) at baseline and also the same scans throughout all patient time points.
2. the average number of target and non-target lesions selected per patient for each LI and BICR readers.
3. whether new lesions were found by LI and/or BICR readers

Part of the analysis required a simultaneous visual comparison of all readers’ assessments in order to understand discrepancies. This task was done by an expert in medical imaging with 10+ years of experience in tumor measurement, supervised by an expert radiologist with 10+ years of experience. The visual comparison consisted of simultaneously displaying the same time point evaluations given by the LI, BICR, and eventually the adjudicator. The expert radiologist was mainly asked to double check any unclear evaluations (e.g., non-measurable lesions are those with equivocal boundaries) or to confirm when complex (e.g. coalescent) target lesions selected by both LI and BICR were the same.

### Evaluations

**Fig. 2:**
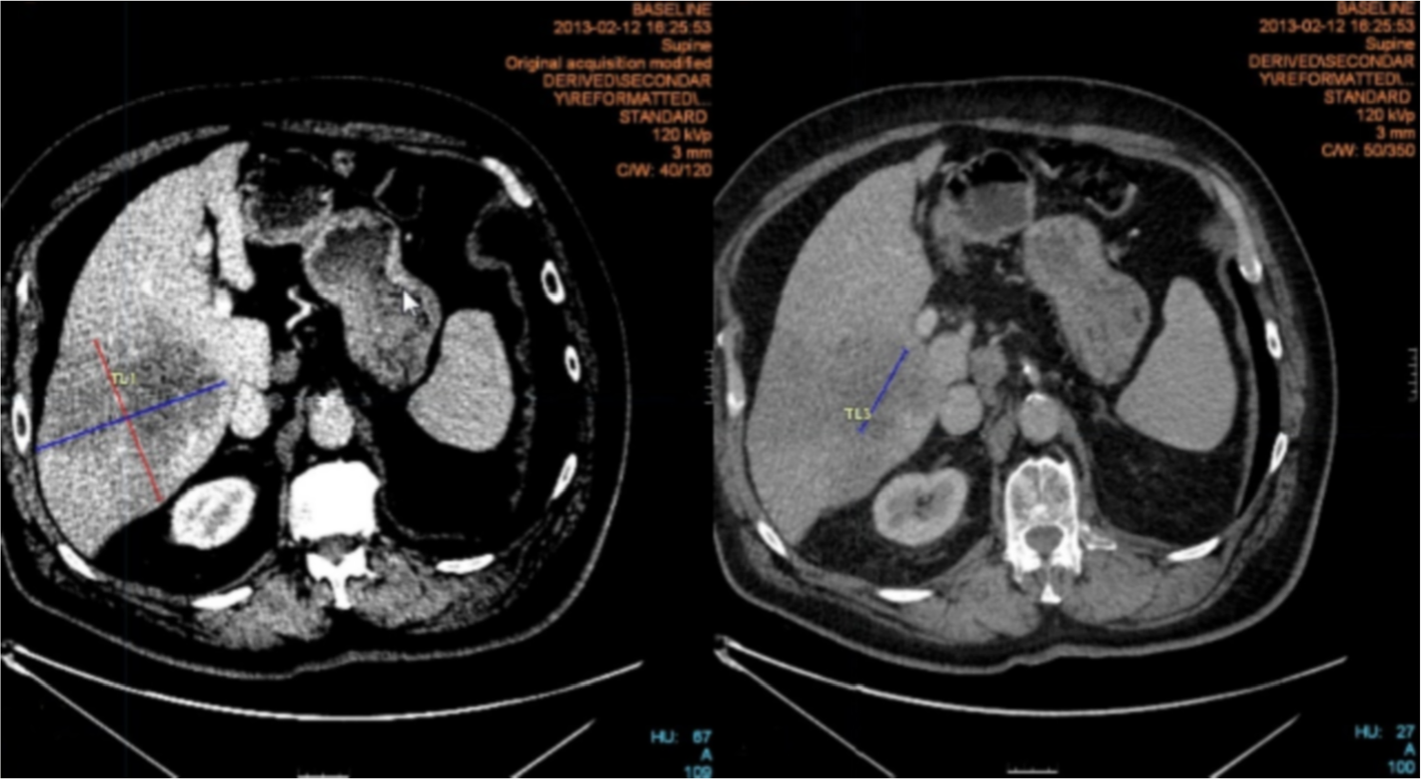
Example of one non-measurable lesion. Both readers targeted the same region of interest, but their perception of tumors’ boundary was. One reader (right) considered the hepatic tumor as coalescent, while the other (left) considered two distinct tumors. The inter-reader proportional difference was 73%. It should be noted that readers enabled different window levels

We reported the proportion of patients with the exact same target lesions selected by LI and BICR, and the number of non-measurable selected lesions (according to Table 1 definition (item 6). Example in Fig. 2). We also analyzed LI and BICR readers discrepancies in reporting new lesions.

Progression-free survival was the primary endpoint of the original trial; therefore, we tested the association between different risk factors (as defined in Table 1) against the requests of adjudication when either the LI or BICR reader declared PD while the other did not. Adjudication was requested at the first time point where a reading discrepancy would occur; discrepancies occurring after the end of treatment were not considered. The design of the original ARD12166 trial did not allow relevant comparisons of PFS drawn respectively from LI and BICR evaluations. When an adjudicator confirmed a PD detected by any of the readers, the patient would then be withdrawn from the trial.

### Statistics

Statistics were processed using R CRAN software (3.0.2, [17]). We tested equality of distributions using non-parametric Wilcoxon statistics. We tested different risk factors (also called “exposure” in biostatistics) likely to trigger adjudications. Association with these different factors were tested by determining the univariate odds ratio with an associated two-tailed *p*-value. An odds ratio significantly different from unity was interpreted as a causation between the tested risk factor and the triggering of adjudication. We compared the proportion of non-measurable lesions among organs using a chi-square test. We also performed a chi-squared test if readers’ assessments which included non-measurable lesions, in the same time, added more target lesions to the tumor burden. We compared the average number of selected target lesions, both when readers selected the exact same target lesions and when they did not, for that we used two-sample t-test.

Our study protocol was reviewed by the Median Technologies medical board and found to be exempt from IRB approval and the need for informed consent. The present study was reviewed and approved by the sponsor. All data were fully anonymized during the original ARD12166 trial, and therefore our study was HIPAA compliant.

## Results

In the original ARD12166 study, 179 patients underwent baseline scans and 147 patients underwent follow-up scans. The adjudication rate at declaring progressive disease per patient was 36.7% (54/147).

### Scans selection

In ARD12166 LI and BICR readers did their assessments using the same baseline scans in 41.3% patients (74/179), and the same follow-up scans in 30.6% patients (45 /147).

### Target lesion selection

**Fig. 3:**
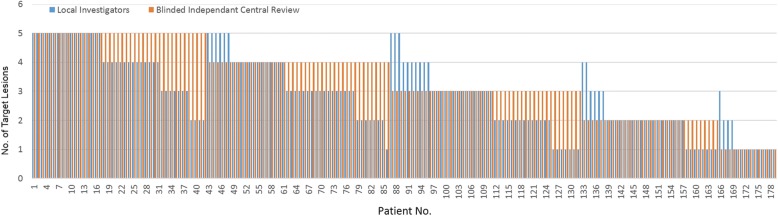
Number of selected target lesions. Number of selected target lesions by LI (blue bars) versus BICR (red bars) readers. Data are ordered first according to the higher number selected by BICR, and then by the higher number selected by the LI. The number of target lesions selected by BICR was significantly higher than that by the LI (pVal< 0.05, Wilcoxon test)

Overall, LI and BICR readers selected a different number of lesions (pVal< 0.005). The average number of target lesions selected by LI, BICR, and the adjudicator were 2.9, 3.4, and 2.5, respectively. Figure 3 presents LI and BICR differences in numbers of selected target lesions per patient.

LI and BICR readers selected exactly the same number of target lesions in 15.6% of patients (28/179) at baseline and in 16.3% of patients (24/147) for the follow-up analysis. When LI and BICR readers selected exactly the same number of target lesions, they selected 1, 2, 3, 4, and 5 lesions in common for 6.1, 4.0, 5.4, 0.7 and 0% of patients, respectively. When LI and BICR had some lesions in common plus other different ones making up their tumor burden, they selected 0, 1… to 4 target lesions in common for 4.5, 33.5, 29, 24 and 9% of patients, respectively.

**Fig. 4:**
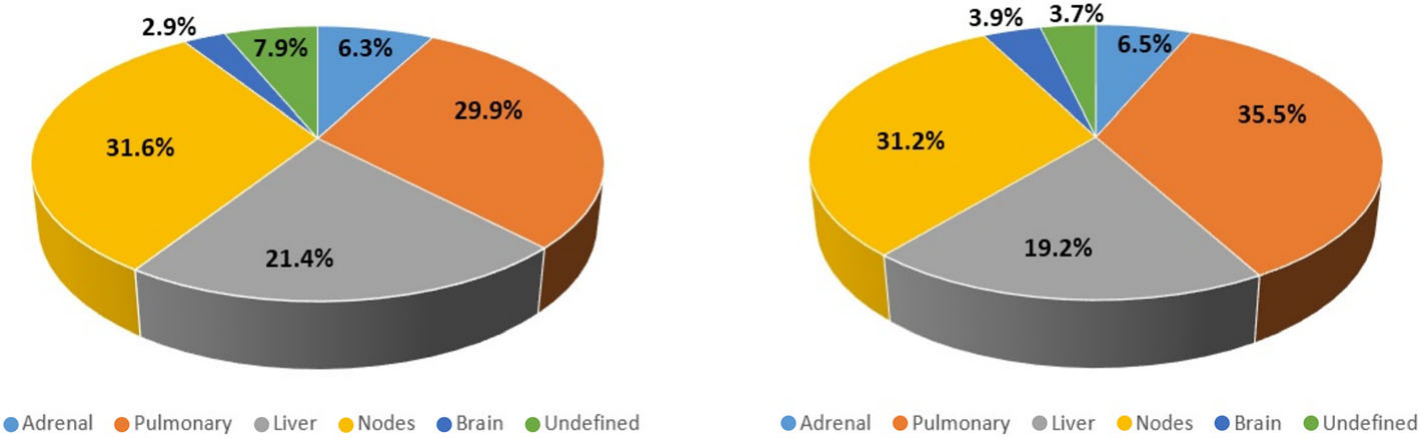
Anatomical sites where target lesions have been selected. Pie chart displaying the proportion of sites where target lesions have been selected. Orange: pulmonary lesions; grey: hepatic lesions; yellow: nodal lesions; light blue: adrenal; navy blue: brain; green: undefined. Undefined lesions are in bone, spleen, pancreas, muscle, and kidney. **Left**: Location of LI target lesion selection. **Right**: Location of BICR target lesion selection

The distribution of selected target lesion sites is reported in Fig. 4. BICR and LI readers preferentially chose pulmonary and nodal lesions rather than hepatic lesions (pVal< 0.05), which is the normal biological distribution in this disease. Some lesions originally labelled as “undefined” were confirmed to be bone, spleen, pancreas, and kidney lesions.

### Non-measurable lesions

We observed that non-measurable lesions (according to Table 1 definitions (item 6)) had either the following features:

- Particularly complex lesions with ill-defined boundaries featuring in some areas, no contrast with surrounding tissues.
- A complex lesion that can be recorded as either two adjacent lesions or a single lobulated one highly textured lesion or lesions with inner structures

We found that the assessment of tumor burden included at least one non-measurable lesion as a target lesion in 18.4% of patients (33/179) at baseline and in 16.3% of patients (24/147) at follow-up. Most non-measurable lesions were labelled as nodal lesions (*n* = 18) by LI and BICR readers, while others were labelled as pulmonary (*n* = 8), hepatic (*n* = 5), and brain (n = 1). One lesion (1/33) was erroneously not labelled. A chi-square test reported no significant difference in the proportion of non-measurable lesions per organ (nodal, pulmonary, hepatic, or brain lesions). We also found that tumor burdens that included non-measurable lesions were also the ones including the more target lesions (pVal = 0.01). Tumor burdens including the same number and same target lesions tended to be tumor burdens with fewer target lesions (pVal< 0.001).

### Selection of non-target lesions

In average, LI and BICR readers selected an equivalent number of non-target lesions (pVal = 0.15). The average number of non-target lesions selected per patient at baseline was 2.1 (SE = 2.4), 1.8 (SE = 1.9), and 0.6 (SE = 0.9) for LI, BICR, and adjudicator readers, respectively.

In 84.1% of patients (58/69), a reader declaring progression of a non-target lesion also reported progression of tumor burden or detection of new lesions. A similar trend was reported in other settings [18]. We also observed that in 70% of cases, when a reader reported progression of non-target lesions, the other reader would report progression of tumor burden or appearance of new lesions.

### Detection of new lesions

The LI, BICR, and adjudicator readers reported 137, 163, and 21 new lesions, respectively in 61, 72, and 17 patients.

LI and/or BICR readers reported at least one new lesion in 61.9% (91/147) of patient follow-up scans. At least one new lesion was detected by LI reader only in 12.9% (19/147) of patient follow-up scans, and by BICR reader only in 20.4% (30/147). The adjudicator confirmed as many new lesions detected by LI readers only (7) as detected by BICR readers only (7), but not in exactly same patients.

### Adjudication rate

In ARD12166, the causes of inter-reader discrepancies at declaring progressive disease were:

- 53.7% (29/54) because one reader declared at least one new lesion,
- 18.5% (10/54) because one reader declared a tumor burden increase of more than 20%.
- 11.2% (6/54) because one reader declared progression of one or more non-target lesions.
- 16.6% (9/54) were due to mixed simultaneous causes of disagreement: one reader declared progression in both tumor burden and in non-target lesions in 7.4% patients; progression of non-target lesions and detection of a new lesion in 5.6% patients and both an increase in tumor burden and the detection of new lesions in 3.7% patients.

### Association between risk factors and adjudication

We tested the six predefined risk factors (shown in Table 1) likely to trigger discrepant readings.

Odds ratios measuring the association between risk factors and adjudications are reported in Table 2.

**Table 2:** Ranking of risk factors likely to trigger adjudications

Risk factors likely to trigger adjudications	Odd ratio[95%CI]	*P* value	*Nb and %* *of occurrence*
Detection of new lesion	1.79 [0.83; 3.9]	0.12	91; 62%
Different image selected at baseline	0.97 [0.47; 2.0]	1.0	85; 57.8%
Different image selected at each time point	0.82 [0.37; 1.79]	0.2	102; 69.4%
Detection of progressive non-target lesion	0.67 [0.31; 1.45]	0.29	52; 35.4%
Selection of non-measurable target lesion	0.52 [0.08; 1.82]	0.25	24; 16.3%
Selection of different target lesions	0.42 [0.16; 1.12]	0.06	123; 83.7%

Risks factors associated with adjudication were assessed in computing the odds ratio with 95% Confidence interval and the corresponding *p* value. Are reported the number of occurrence of these risks factors per patients and the proportion they represented in our dataset

We top ranked the **detection of new lesions** as one of the major issues with an odd ratio of 1.79 as confirmed by reviews of cases.

The **selection of different scans** by LI and BICR was not associated with higher adjudications rate.

No association was found between the declaration of **progressive non-target lesions** and adjudications.

We found no association between the selection of **non-measurable lesions** and adjudications even if non-measurable lesion measurements frequently exhibit very large differences.

We compared tumor burden including less or exactly 3 target lesions against more than 3 targets. We showed that readers tend to proportionally select more non-measurable tumors when more target lesions can be selected (pVal = 0.017), therefore when readers measure large tumor burdens.. Consequently, tumor burden differences due to non-measurable tumors were largely averaged by the other well-defined target measurements.

Lastly, we found a close inverse association between the number of adjudications and the fact that readers selected **different target lesions** (pVal = 0.06).

## Discussion

Our study showed a discrepancy rate between LI and BICR of 36.7% at declaring PD per patients. Similar outcomes were reported by others such as Ford et al., who identified discrepancy rates (24%–29%). Another trial reported a rate of 38.6% [7]. In our study, we determined that declaring one or more new lesions was the leading cause of inter-reader disagreement and was responsible for more than half of adjudications. Disagreements in the change of tumor burden triggered about 20% of adjudications, and disagreements in declarations of progressive non-target lesions triggered just over 10% adjudications. In a different context, a general analysis from K. Borradaile et al. [19] reported that 37% of discrepancies involved the selection of target lesions and 30% involved the perception of new lesions. In that particular study, the number of discrepancies due to new lesions was also high; however, we can reasonably hypothesize that the proportion of discrepancies due to various risk factors may depend on study settings, modalities, and diseases.

The lack of reliability at detecting new lesion should be investigated as it could come from imperfect compliance to criteria rules or from the misinterpretation of rules by readers or from unspecific RECIST guidance. Indeed, along with the revision of RECIST guidelines, dedicated recommendations for assessing lymph nodes were published [20]. Unfortunately, these authors did not provide any specific guidance for the identification of new nodal lesions, neither for previously visible and growing nor for previously not-visible nodes. Another important point is to determine if, within the dual-reading paradigm, the LI and BICR readers have equivalent sensitivity in detecting new lesions.

While many progressive non-target lesions were reported in this study, they had a limited impact on adjudication rates because they were frequently associated with the simultaneous detection of new lesions or with an increase in the measured tumor burden as observed in 58/69 (84.1%) patients. However, the role of non-target lesions was not negligible, as they accounted for more than 10% of adjudications.

We wanted to measure the strength of association between requests for adjudication and several other risk factors that are not markedly addressed in the literature. We found no issue related to LI/BICR selecting different imaging scans. Therefore, it would appear that any attempt to constrain readers to analyze exactly the same scans at baseline and at each time point would likely bring no improvements in the adjudication rates.

Although the presence of non-measurable lesions was thought to degrade the reliability of tumor burden assessment [21], we surprisingly found an inverse association between non-measurable lesion presence and adjudications. We also found that readers tended to select non-measurable lesions when they selected a larger number of target lesions. This counter-intuitive inverse association may be explained by the findings of Schwartz et al. [15], who observed that increasing the number of selected tumors could average out measurement errors.

Similarly, we found a very near inverse association between adjudication rates and selection of different target lesions between readers. Here, contrary to other studies [22], we found that the likelihood of triggering adjudication was higher when 2 readers would select exactly the same target lesions. We showed that tumor burden assessments based on exactly the same number and same target lesions were primarily done in patients with minor tumor spread; this happens when readers have fewer lesions to choose from in patients with a smaller disease burden. The limited number of detected lesions may preclude any error averaging effect.

Generally speaking, the reliability of assessments is less affected by either non-measurable lesions or randomness in lesion selection when tumor burden involves more than 2 target lesions. According to trial inclusion criteria, only patients with advanced disease were enrolled, therefore most patients had several target lesions.

We found that LI and BICR readers selected different numbers of target lesions per patient. This difference was small, and it is difficult to establish whether it had a significant impact on the precision of assessments and on the adjudication rate. For future investigations, it would be interesting to report the difference between selected target lesions and the number displayed in the scans, per reader and per patient. This would be a valuable metric for optimal assessment, assuming that a robust ground truth of target lesions could be obtained.

The latest version of RECIST guidelines (RECIST 1.1) fixed several issues, however further improvements are needed to avoid variability due to new lesions declaration. Assessment standardization can be improved by advanced training and stringent annotation quality control [23, 24]. Improvements from technology and software in particular can also be expected as Machado et al. [25] reported an improvement, from 52 to 78%, of the concordance in radiology reports. Several groups are investigating the use of alternative quantitative imaging biomarkers such as tumor volumetry [26], for a volume-based quantification that may be able to give more sensitive and reliable assessments. Furthermore, semi-automatic segmentations systems reportedly improve reliability of measurements [27] by minimizing user interaction.

### Our study has some limitations

First, the ARD12166 trial specific settings and the specific disease (SCLC) which is not the most common type of lung cancer, limit the generalization of results.

Secondly, we did not consider the possibility that a reader could make mistakes while reporting findings that were not clinically pertinent (e. g., splenosis, adrenal adenoma, benign liver lesions, etc). To be perfectly rigorous, a board-certified panel of experts would have to systematically confirm all annotations. However, variability in data selection was the endpoint of our study, the clinical appropriateness of findings was not defined as a risk factor for this study, but it would be particularly interesting to consider in future studies.

Thirdly, in the ARD12166 trial, readers had different level of expertise, even if all readers were properly trained and identified as qualified readers. Measuring the impact of readers’ expertise clearly deserve further investigations [28] as a complete analysis of readers training could potentially explain additional reading discrepancies.

Lastly, our definition of non-measurable lesion was subjective (see Table 1), therefore our finding that 18.4% of lesions were classified as non-measurable can probably be challenged.

## Conclusion

Based on the importance of risk factors, the logical next steps would be to: 1) analyze more specifically the process of new lesion declaration and to 2) improve tumor burden assessment.

Future clinical trials will certainly benefit from new technologies in reducing human interactions and involving more automated routines to standardize measures.

## Abbreviations

BICR: Blinded independent central review
CRO: Clinical research organization
CT: Computerized tomography
ECOG: Eastern Cooperative Oncology Group
IRB: Institutional review board
LI: Local investigators
MRI: Magnetic resonance imaging
PD: Progressive disease
RECIST: Response evaluation criteria in solid tumors
SCLC: Small cell lung cancer
SE: Standard Error
